# Genome-Wide Identification of *BjNCED* Gene Family and Expression Analysis in Response to ABA, Salt, and Low-Temperature Stresses in *Brassica juncea*

**DOI:** 10.3390/plants15152372

**Published:** 2026-08-01

**Authors:** Xinwen Wang, Hangli Li, Weiming Gong, Yuekun Han, Yuhang Chen, Zhe Zeng, Dawei Zhang, Jinfeng Wu, Xiaolan Liu, Lili Liu, Yang Xu, Mingli Yan, Dinggang Zhou

**Affiliations:** 1 Hunan Key Laboratory of Economic Crops Genetic Improvement and Integrated Utilization, School of Life and Health Sciences, Hunan University of Science and Technology, Xiangtan 411201, China; wangxinwen2001@163.com (X.W.); lihangli2024@163.com (H.L.); gwm0258@163.com (W.G.); hanyuekun2000@163.com (Y.H.); cyh611720@163.com (Y.C.); zengzhe2306@163.com (Z.Z.); zhangdawei.hnust@foxmail.com (D.Z.); wujinfeng.hnust@foxmail.com (J.W.); liuxiaolan1018@126.com (X.L.); lilyliu@hnust.edu.cn (L.L.); xu10022020@163.com (Y.X.); 2 Hunan Engineering Research Center of Lotus Deep Processing and Nutritional Health Sciences, Hunan University of Science and Technology, Xiangtan 411201, China; 3 Crop Research Institute, Hunan Academy of Agricultural Sciences, Changsha 410125, China; ymljack@126.com

**Keywords:** *Brassica juncea*, *NCED* gene family, abscisic acid (ABA), cold stress, salt stress

## Abstract

NCED proteins play a critical role in drought, salt, and cold stress responses through ABA biosynthesis in plants. However, little information is currently available regarding the *NCED* gene family in *Brassica* species. Twenty-six putative *BjNCED* genes were identified in the genome of *Brassica juncea*, and found to be distributed on 15 chromosomes. Phylogenetic analysis suggested that these members could be classified into six subfamilies. The putative cis-elements were identified in the promoter regions of these *BjNCED* genes, and thought to be related to phytohormones, light, and abiotic stress responses. qRT-PCR analysis of five genes (*BjNCED1*, *BjNCED4*, *BjNCED7*, *BjNCED16* and *BjNCED18*) revealed that, under salt stress, *BjNCED16* and *BjNCED18* showed transient induction, whereas exogenous ABA produced gene-specific and time-dependent expression patterns, including pronounced induction of *BjNCED16* at 24 h. Low-temperature treatment strongly induced four of the five examined genes at 6 h. Analysis of the *BjNCED* genes in this study provides a valuable foundation for future investigations into the functional roles of the *BjNCED* family in response to growth, development and stress.

## 1. Introduction

Given the continued escalation of global climate change and associated environmental stresses, deciphering how plants perceive, adapt, and respond to external stressors has become a critical area of investigation in plant biology [1]. Abscisic acid (ABA) content, salt stress, and low-temperature tolerance are the key factors that enable plants to adapt to environmental fluctuations, which directly influence survival, growth, and development in plants [2,3,4]. Therefore, understanding how gene expression is regulated in plants under abiotic stress conditions is critically important for advancing plant stress biology, and can reveal plant adaptability and assist in the cultivation of stress-resistant varieties.

*Brassica* species, belonging to the family Brassicaceae (also known as crucifers), represent a group of major oil and vegetable crops with global agricultural significance [5,6]. As an annual herbaceous species, *Brassica juncea* is widely planted across China. It includes a broad spectrum of varieties, most of which possess desirable traits such as high biomass accumulation, rapid growth, and strong environmental tolerance, thereby supporting several cropping cycles annually [7]. These plants, including *B. juncea*, respond to environmental stresses (low temperature and salinity) and hormonal signals (ABA) through coordinated physiological and biochemical adjustments, including ion homeostasis and reactive oxygen species (ROS) detoxification [8,9]. Low temperatures alter plant metabolism and physiology, affecting Ca^2+^, sugars, ROS, and hormone levels [10,11,12,13]. The extent of these alterations contributes to the observed variation in plant cold tolerance. A substantial body of evidence indicates that exogenous ABA significantly increases cold stress tolerance in various plant species, including *Stylosanthes guianensis* [14], *Actinidia chinensis* Planch [15], and *Triticum aestivum* L. [16]. Both salt and drought stress can perturb the redox equilibrium within plant cells, which in turn triggers secondary oxidative stress and results in the excessive generation of ROS [17]. Such damage to biomacromolecules caused by oxidative stress, including proteins and lipids, further impairs cellular functions and consequently suppresses plant development. Notably, the fresh weight of seedlings treated with 225 mM NaCl emerged as a suitable indicator of the variation in saline response across the *B. juncea* genotype [18]. Exogenous ABA can partially or fully mitigate salt toxicity in rice seedlings and restore most salt-induced physiological alterations [19]. Therefore, elucidating the regulatory mechanisms of gene expression under abiotic stress is important for enhancing stress resistance and ensuring production quality in *B. juncea*.

9-cis-epoxycarotenoid dioxygenase (NCED) serves as a key enzyme in plants, playing a rate-limiting role in the biosynthesis of ABA by catalyzing the specific cleavage of 9-cis-epoxycarotenoids to yield xanthoxin, an essential precursor of ABA [20,21,22]. The first identified *NCED* gene, designated *VP14*, was discovered in maize (*Zea mays*) [23]. Through transcriptome analysis of drought-stressed *Populus davidiana*, *NCED* was found to be a crucial regulator conferring drought tolerance [24]. In *Oryza sativa*, *OsNCED5*, a chloroplast-localized enzyme involved in ABA biosynthesis, exhibited strong transcriptional induction in response to cold stress, and exogenous ABA was capable of restoring cold tolerance in *OsNCED5* mutants [25]. Overexpression of *OsNCED3* conferred enhanced drought tolerance and resulted in increased ABA accumulation [26]. In *Arabidopsis thaliana*, the three *NCED* genes (*AtNCED5*, *AtNCED6*, and *AtNCED9*) were primarily responsible for ABA production in seeds under developmental regulation, thus orchestrating the establishment of dormancy and embryo maturation [27]. The transcripts of *AtNCED2* and *AtNCED3* were highly expressed in the roots, and the proteins encoded by these genes participate in ABA biosynthesis, thus playing a regulatory role in lateral root growth [27]. In *Lonicera japonica* Thunb., *LjNCED*1 and *LjNCED*2 were markedly up-regulated under salt stress [28].

At present, NCED genes have been identified in *Persea americana* [29], *A. thaliana* [27], *Phaseolus vulgaris* [30], *Vigna unguiculata* [31], *Z. mays* [32], and other plants [33]. The changing global climate means that plants face all kinds of threats in the natural environment. With rapid advances in plant genomics technology and molecular biology research, scientists use modern molecular biotechnology to understand the stress resistance mechanism of crops at the molecular level, bringing new opportunities for crop resistance breeding. While the *NCED* family has been characterized in *Brassica napus*, no comprehensive investigation has been conducted on the *BjNCED* members in *B. juncea* with respect to their responses to hormonal and environmental cues. In the present study, we performed a genome-wide identification of the *BjNCED* genes and characterized their expression profiles under ABA treatment and salt and cold stress. This systematic characterization, combined with quantitative real-time PCR (qRT-PCR) analysis, aims to reveal the regulatory patterns of *BjNCED*s in stress adaptation. This work will accelerate the genetic improvement of stress-tolerant *B. juncea* varieties.

## 2. Results

### 2.1. Identification and Analysis of the BjNCED Genes in B. juncea

Initially, 31 candidate genes were retrieved, and after domain validation, 26 *BjNCED* genes were confirmed as BjNCED family members. A comprehensive search of the *B. juncea* whole-genome database revealed 26 *BjNCED* genes, which were subsequently designated as *BjNCED1*–*BjNCED26*. To gain insight into the distinct characteristics of each *BjNCED* member, we systematically evaluated their physicochemical properties, such as the number of amino acids, molecular weight (MW), instability index, isoelectric point (pI), grand average of hydropathy (GRAVY), and predicted subcellular localization (Table 1). The protein length of the 26 BjNCED proteins spanned from 314 to 881, and the MW ranged from 34,587.57 Da to 98,201.3 Da. Analysis of the pI revealed a broad range from 5.32 to 9.44 among the BjNCED proteins. Of these, 25 were acidic (pI < 7) and the remaining BjNCED17 was alkaline (pI > 7). Instability index analysis revealed that proteins with values less than 40 are considered stable. Accordingly, the seven members of the *BjNCED* family (*BjNCED5*, *BjNCED9*, *BjNCED13*, *BjNCED15*, *BjNCED17*, *BjNCED20* and *BjNCED21*) fell below this threshold and were thus designated as stable proteins. Analysis of the GRAVY scores, which ranged from −0.363 to −0.169, revealed that all BjNCED proteins are hydrophilic. Predicted subcellular localizations indicated that the BjNCED proteins are targeted to various organelles: the majority localize to the chloroplast (21 proteins), followed by the cytosol (2 proteins), mitochondrion (2 proteins), and a single protein in the nucleus.

### 2.2. Phylogenetic Analysis of BjNCED Genes

**Figure 1:**
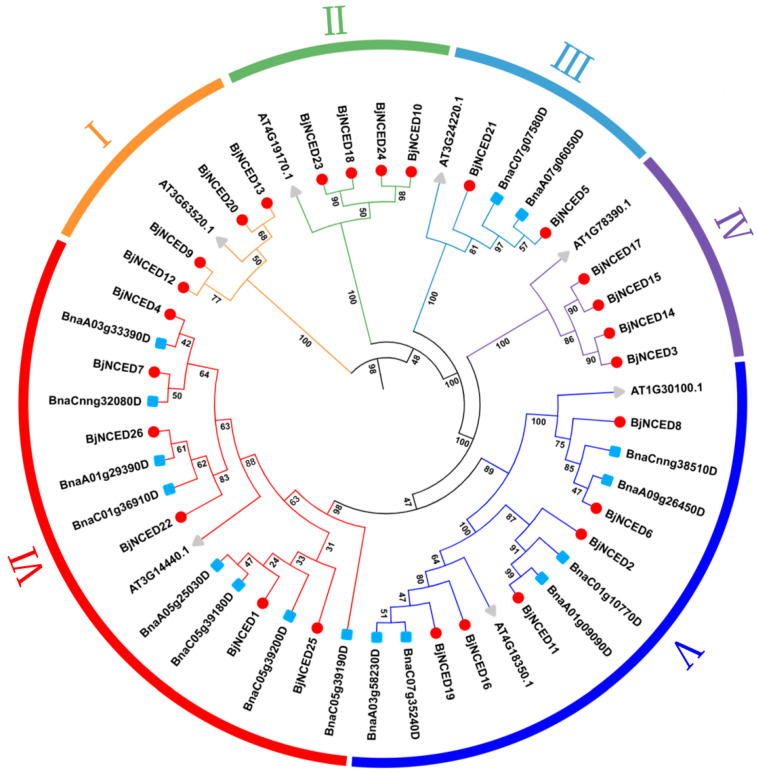
A maximum likelihood phylogenetic tree of the *NCED* family in *B. juncea*, *B. napus,* and *A. thaliana*. The unrooted maximum likelihood tree was constructed in MEGA 12 using the ClustalW-aligned full-length protein sequences. Bootstrap analysis was performed with 1000 replicates, and gaps were handled by partial deletion. The optimal amino acid substitution model was LG, with gamma-distributed rate heterogeneity (+G) and a proportion of invariant sites (+I). The tree resolved 49 NCED proteins into six major clades (designated Groups I, II, III, IV, V, and VI). Circles: *B. juncea*; squares: *B. napus*; triangles: *A. thaliana*.

To investigate the phylogenetic relationships of NCED proteins from *B. juncea*, *B. napus*, and *A. thaliana*, a phylogenetic tree was constructed based on 49 protein sequences. Sequence similarity analysis classified the members into six groups (Group I to Group VI) (Figure 1). Six BjNCED proteins (BjNCED1, BjNCED4, BjNCED7, BjNCED22, BjNCED25 and BjNCED26), one AtNCED protein (AT3G14440.1), and eight BnNCED proteins (BnaA05g25030D, BnaC05g39200D, BnaC01g36910D, BnaA01g29390D, BnaA03g33390D, BnaC05g39180D, BnaC05g39190D and BnaCnng32080D) were categorized into Group VI. Six BjNCED proteins (BjNCED2, BjNCED6, BjNCED8, BjNCED11, BjNCED16 and BjNCED19), two AtNCED proteins (AT1G30100.1 and AT4G18350.1), and six BnNCED proteins (BnaC07g35240D, BnaA03g58230D, BnaA01g09090D, BnaC01g10770D, BnaA09g26450D and BnaCnng38510D) were classified into Group V. Four BjNCED proteins (BjNCED3, BjNCED14, BjNCED15 and BjNCED17) and one AtNCED protein (AT1G78390.1) were classified into Group IV. Two BjNCED proteins (BjNCED5 and BjNCED21), one AtNCED protein (AT3G24220.1), and two BnNCED proteins (BnaC07g07580D and BnaA07g06050D) were classified into Group III. Four BjNCED proteins (BjNCED10, BjNCED18, BjNCED23 and BjNCED24) and one AtNCED protein (AT4G19170.1) were classified into Group II. Four BjNCED proteins (BjNCED9, BjNCED12, BjNCED13 and BjNCED20) and one AtNCED protein (AT3G63520.1) were classified into Group I.

Notably, the close clustering of several BjNCED proteins with *Brassica* homologs is consistent with the conservation of ancestral NCED lineages and subsequent retention following polyploidization.

### 2.3. Phylogenetic Analysis of BjNCED Genes and Analysis of Their Gene Structure and Conserved Motifs

**Figure 2:**
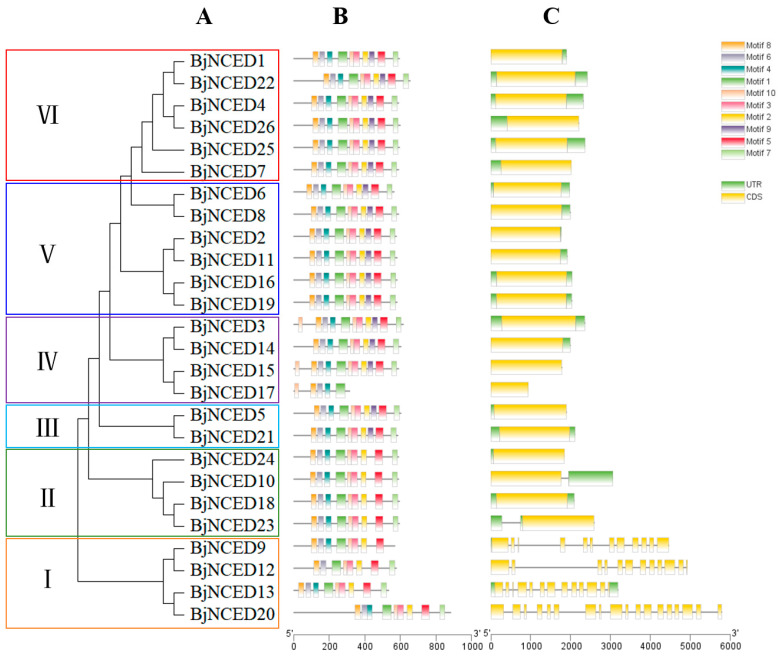
Gene structure and conserved motifs of the *BjNCED* family in *B. juncea*. (**A**) Phylogenetic tree showing in four groups (I–VI). (**B**) Ten conserved motifs (colored boxes) in BjNCED proteins. (**C**) Gene structure distribution of the *BjNCED* gene family in *B. juncea.*

An unrooted phylogenetic tree was generated using sequence alignments of 26 *BjNCED* genes (Figure 2A). The 26 *BjNCED* genes were classified into six groups: Groups V and VI comprised the majority (12 genes), Groups I, II, and IV each contained four genes, and Group III contained two genes.

The gene structure diagrams of the *BjNCED* genes were visualized with TBtools software (Figure 2C). There were significant differences in the numbers and lengths of the 26 *NCED* introns and untranslated regions (UTRs). Five *BjNCED* genes contained one 5′-UTR (*BjNCED5*, *BjNCED7*, *BjNCED9*, *BjNCED24* and *BjNCED26*). One *BjNCED* gene contained two 5′-UTRs (*BjNCED23*). Five *BjNCED* genes contained one 3′-UTR (*BjNCED1*, *BjNCED2*, *BjNCED8*, *BjNCED11* and *BjNCED14*). Four *BjNCED* genes did not possess any UTR region (*BjNCED15*, *BjNCED17*, *BjNCED12* and *BjNCED20*), while the remaining eleven each contained a single 5′-UTR and a single 3′-UTR. Moreover, significant variation in both the number and the spatial arrangement of exons was observed among the six groups.

The results revealed a total of 10 conserved motifs distributed in BjNCED protein sequences (Figure 2B). Minor changes were observed in the lengths of the motifs, suggesting that conservation among the *BjNCED* family members is relatively high. Further analysis of the distribution of these conserved motifs demonstrated that most of the BjNCED family members contained 10 conserved motifs. However, some genes missed a certain number of conserved motifs. BjNCED17 had the least conserved motifs, indicating that its functions might be more differentiated than those of other members. In addition, we found that 26 genes all had two conserved domain sequences—MIAHPKxDP and HDFAITE—unique to the NCED family and four conserved histidine residues, with domain coverage across the BjNCED proteins ranging from 62.20% to 100%. (Figure S1 and Table S4).

### 2.4. Chromosomal Localization and Collinearity of BjNCED Gene Family

**Figure 3:**
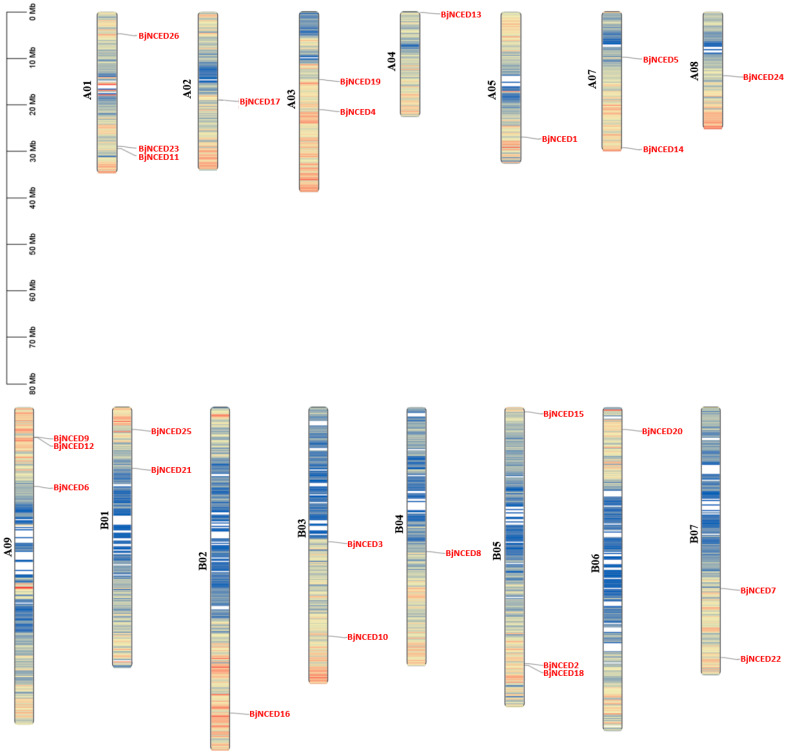
Collinearity analysis of the 26 *BjNCED* genes in *B. juncea*. Left scale bar: chromosome length (Mb). Color gradient represents gene density, with darker colors corresponding to higher gene density.

The 26 *BjNCED* genes were not uniformly distributed; instead, they were mapped to 15 different chromosomes of the *B. juncea* genome (Figure 3). Seven chromosomes contained one *BjNCED* locus (A02, A04, A05, A08, B02, B04, and B06), four chromosomes contained two loci (A03, A07, B01, B03, and B07) and three chromosomes contained three loci (A01, A09, and B05) (Figure 3).

**Figure 4:**
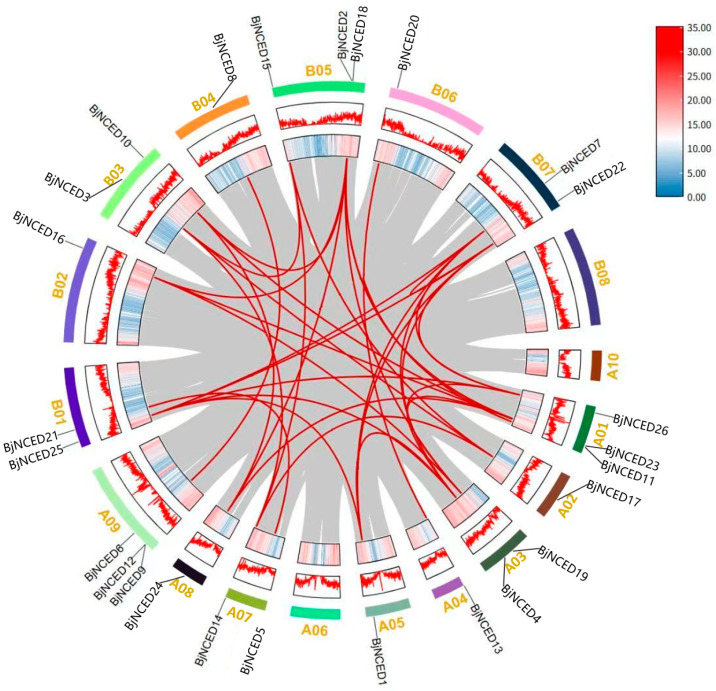
Collinearity analysis of the 26 *BjNCED* genes in *B. juncea*. Gray lines represent collinear relationships between all genes across the genome, red lines highlight segmental duplication gene pairs within the *BjNCED* family. Chromosomes labeled “A01–A10” and “B01–B08” correspond to the A and B subgenomes, respectively.

One tandem duplication event was identified among *BjNCED* family members through evolutionary analysis (*BjNCED9* and *BjNCED12*). In addition, 36 pairs of segmental duplication events were detected, involving 24 genes (excluding *BjNCED9* and *BjNCED12*). This suggests that both tandem and segmental duplications contributed substantially to the evolutionary expansion of the *BjNCED* gene family (Figure 4 and Table S1).

### 2.5. Analysis of Cis-Regulatory Element of BjNCED Promoters in B. juncea

**Figure 5:**
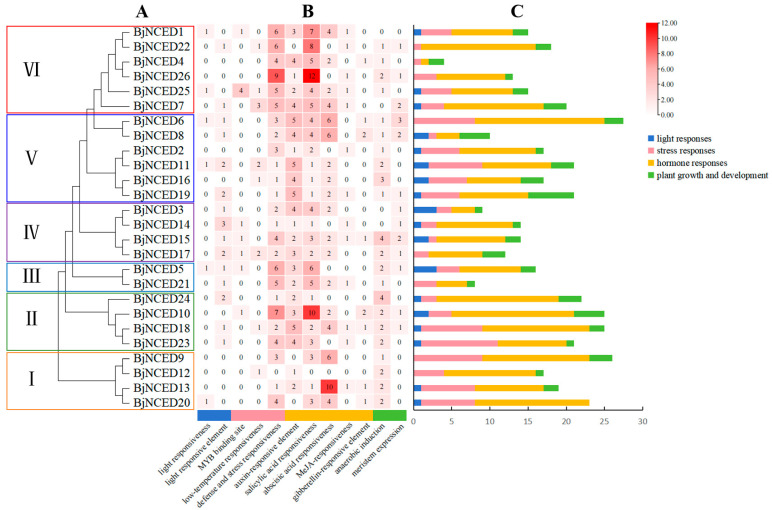
Cis-element profiles of the *BjNCED* genes. (**A**) Phylogenetic tree showing Groups I to VI. (**B**) Cis-element counts: numbers in boxes indicate numbers; darker red corresponds to higher numbers; cis-element types are shown at the bottom. (**C**) The four different colors indicate the number of cis-acting element functions.

Cis-element analysis of the 26 BjNCED promoters identified diverse regulatory motifs associated with abiotic/biotic stress responses, light signal transduction, and phytohormone signal cascades, as well as fundamental plant growth and developmental pathways (Figure 5). Within growth- and development-related regulatory regions, two characteristic cis-acting motifs were captured: one drives specific gene expression in meristematic tissues, while the other confers transcriptional activation under anaerobic environmental conditions. A total of five distinct hormone-responsive motifs were annotated, corresponding to signal perception of abscisic acid, methyl jasmonate, auxin, gibberellin, and salicylic acid, indicating that these *BjNCED*s might be modulated by multiple phytohormone signaling networks. Three functional motifs linked to stress tolerance were also detected, consisting of cold-inducible fragments, MYB transcription-factor binding sites, and universal defense-stress responsive sequences. In addition, the presence of light-responsive cis-regulatory motifs suggested that transcription can be actively regulated by ambient light signals throughout plant life cycles.

### 2.6. Phenotype Analysis, Expression Analysis, and qRT-PCR Analysis

**Figure 6:**
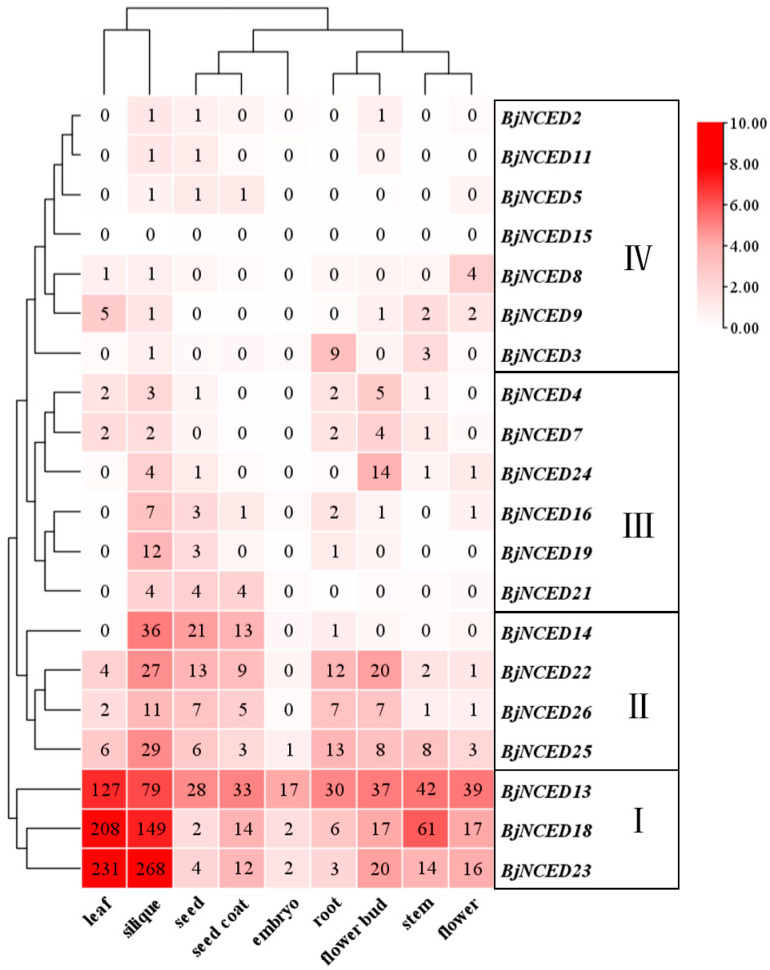
Expression profiles of *BjNCED* genes across different tissues of *B. juncea*. Red: high expression; white: low expression. Numbers indicate expression levels. Classes I to IV represent the three phylogenetic subgroups. Color legend labeled with logarithmic transformation.

Nine tissues of *B. juncea* were analyzed from TPM transcriptomic expression unit data to obtain the tissue-specific expression patterns of *BjNCED* genes, including silique, leaf, stem, embryo, flower, seed, seed coat, root, and flower bud (Figure 6). A hierarchical clustering heatmap clearly separated these *BjNCEDs* into four expression clusters (Classes I, II, III, and IV) with drastically divergent tissue expression signatures.

The results revealed that the most highly expressed genes in each tissue belonged to Class I, among which *BjNCED23* displayed the greatest transcript abundance in silique and leaf, followed by strong tissue-preferential expression of *BjNCED13* and *BjNCED18*. In contrast, three genes in Class II showed high expression in almost every tissue: *BjNCED22* had high expression in silique and flower bud, *BjNCED25* had high expression in silique, and *BjNCED*14 had high expression in silique and seed. The remaining seven genes (*BjNCED4*, *BjNCED7*, *BjNCED15*, *BjNCED8*, *BjNCED2*, *BjNCED5* and *BjNCED11*) in Classes III and IV maintained extremely weak expression signals in every sampled tissue. This low expression level suggests that these genes may have only limited transcriptional roles in the examined organs under normal developmental conditions; however, their potential functions in other tissues, developmental stages, or under stress conditions cannot be excluded.

**Figure 7:**
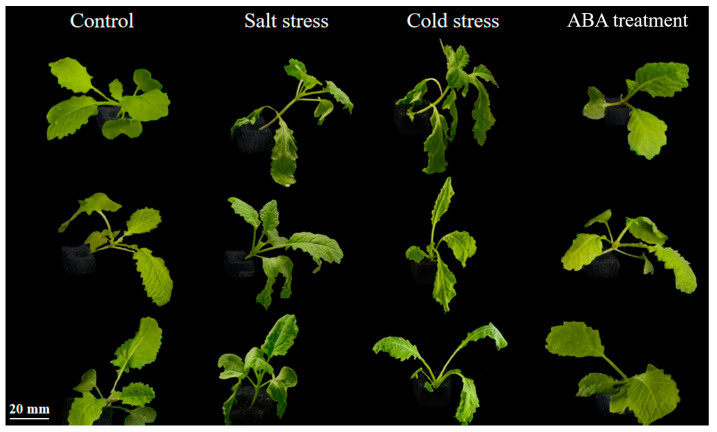
Phenotype of Purple Leaf Mustard seedlings under salt, ABA, and cold treatments after 48 h. Control: seedlings without any treatment at 48 h; Salt stress, Cold stress, and ABA treatment: 48 h salt, cold, or ABA treatment on *B. juncea* seedlings. Scale bar = 20 mm.

We analyzed the phenotype of Purple Leaf Mustard under different stress treatments after 48 h (Figure 7). Under salt stress and cold stress, the Purple Leaf Mustard showed an obvious wilting state, but it did not show wilting under ABA treatment.

**Figure 8:**
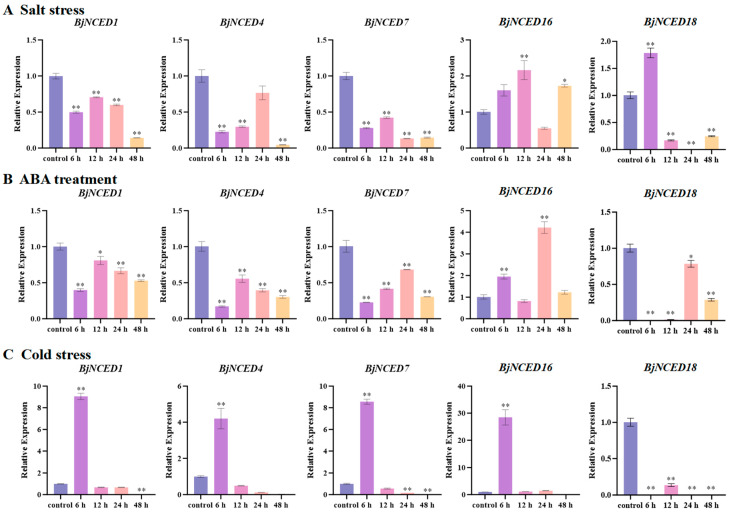
Expression levels of *BjNCED* genes under salt and low-temperature stresses and ABA treatments. (**A**) Relative expression patterns under 150.0 mM NaCl. (**B**) Relative expression patterns under 0.10 mM ABA spray. (**C**) Relative expression patterns under cold stress conditions at 4 °C. Expression levels at 6, 12, 24 and 48 h are shown relative to the control (0 h). The error bars mean ± SE. ** *p* < 0.01; * *p* < 0.05.

For expression pattern analysis, five representative members were chosen under salt, ABA, and low-temperature stress conditions through qRT-PCR (Table S3 and Figure 8). Most of the examined *BjNCED* genes were down-regulated under salt stress, and *BjNCED16* was significantly up-regulated at the early time points of 6 h and 12 h, while *BjNCED18* was significantly up-regulated at the early time point of 6 h. Under ABA treatment, *BjNCED* gene expression fluctuated greatly, and *BjNCED7* exhibited initial up-regulation followed by subsequent down-regulation, indicative of a transient response. Low temperature induced *BjNCED* genes significantly, with the exception of *BjNCED18*; the expression of the remaining genes (*BjNCED1*, *BjNCED4*, *BjNCED7* and *BjNCED16*) increased significantly (with expression levels significantly higher than those of the control) at 6 h of treatment. In summary, the expression of *BjNCED* genes displayed diverse and gene-specific patterns under the three stress conditions tested, with some genes showing rapid down-regulation and others exhibiting sustained or delayed induction. These data suggest that individual *BjNCED* members may contribute differentially to stress responses, and the observed transcriptional profiles offer valuable clues for future functional studies.

## 3. Discussion

ABA plays important roles in plant growth and development, and stress response conditions [34,35]. Exogenous ABA significantly enhances cold stress tolerance in various plants and partially or completely alleviates salt toxicity in seedlings and restores their salt-induced physiological changes [14,19]. Salt stress triggers drought-like adaptive responses, including reduced transpiration and stomatal closure [36]. Prolonged low-temperature stress injures plants and impairs crop production by suppressing seed germination, growth, yield, and quality [37,38]. Nonetheless, many plants can enhance freezing tolerance via cold acclimation, which involves prior exposure to low, non-freezing temperatures [39]. As a rate-limiting factor in the ABA biosynthesis pathway, *NCED* critically contributes throughout all stages of plant growth and development, as well as in diverse abiotic stress responses [40,41]. The function of the *BjNCED* family has not been well-characterized, especially in relation to yield. Here, we performed a genome-wide identification of this family using bioinformatics. Meanwhile, we analyzed the expression of representative *BjNCED* genes under different stresses.

This study aimed to identify and analyze the NCED gene family in *B. juncea*, focusing on their roles in ABA biosynthesis and abiotic stress responses. A total of 26 *BjNCED* genes were identified based on the *B. juncea* genome database. More *NCED* genes were identified than have been found in tomato (11 *SlNCEDs*) or *A. thaliana* (9 *AtNCEDs*) [27,33]. This significant expansion is probably related to the formation of the allotetraploid (AABB) genome in *B. juncea*, which provides a large number of duplicate copies for gene families, thus providing raw materials for functional differentiation and adaptive evolution. Phylogenetic analysis showed that the above 26 *BjNCED* genes could be classified into six subfamilies, each with different structural and functional characteristics. Each branch of the phylogenetic tree contained homologous NCED proteins of *A. thaliana*, indicating that the NCED subfamily had high conservation in evolution. Subcellular localization analysis revealed that the majority of BjNCED proteins are targeted to the chloroplast, consistent with their role in ABA biosynthesis, a finding also reported in *Prunus avium* L. [42].

The cis-element analysis indicated that among the 26 *BjNCED* genes, 18 genes contained light-responsive elements and all genes contained stress-responsive elements, and all except *BjNCED1* carried elements related to growth and development. Moreover, various hormone-responsive cis-elements were detected in the promoter regions. This structural feature is highly consistent with the composition of the *NCED* promoters in rice, peanut, and other species, further supporting the potential conserved regulatory mechanism of the *NCED* gene in plant stress responses [43,44]. Integrating cis-element prediction and qRT-PCR transcription profiles, individual *BjNCED* members were shown to exhibit divergent transcriptional trends under abiotic treatments. Notably, most tested *BjNCED* genes were suppressed under salt stress, while only a subset (*BjNCED16* and *BjNCED18*) displayed transient up-regulation. Collectively, promoter cis-regulatory signatures and expression data suggest that different *BjNCED* genes may possess distinct transcriptional regulatory potential upon environmental stimulus, rather than indicating uniform positive stress responsiveness across the entire gene family.

The processes of gene duplication and loss are fundamental drivers of evolutionary change in species, not only generating functional novelty but also promoting the proliferation and diversification of gene families [45]. The *NCED* gene family has been well established in showing that gene duplication and functional divergence constitute the major evolutionary routes for generating novel genes and broadening gene functional repertoires [46]. A total of 36 pairs of segmental duplication events and one tandem duplication event were detected in the *BjNCED* gene family, revealing that segmental duplication predominantly drove the expansion of the *BjNCED* family throughout evolution. This pattern is different from the tandem repeat expansion mechanism of *SlNCED*s in tomato, reflecting the differences in evolutionary strategies of *NCED* families among different species [33].

We analyzed the expression patterns of the *BjNCED* family to characterize their transcriptional responses to salt stress, ABA, and low temperatures. Different *BjNCED* genes responded differently to the same stress, and the same gene also responded differently to different stresses (NaCl, ABA, or low temperature). For example, *BjNCED16* was transiently up-regulated under salt stress, exhibited an initial increase followed by a decrease upon ABA treatment, and showed transient induction under low temperature. These results indicate that the *BjNCED* gene may be as involved in the resistance response to salt stress, ABA treatment, and low-temperature stress as in peanut and rice [43,44]. This is in line with other findings, which reported that *NCED* genes play a role in the salt stress response through maintaining osmotic balance [17]. *BjNCED18* peaked at 6 h under salt stress, and then declined. This pattern may be related to negative feedback regulation. In addition, *BjNCED18* constituted an outlier under cold stress relative to other paralogs. This divergent expression may stem from differences in cis-regulatory sequences; one possibility is that the *BjNCED18* promoter lacks essential cold-responsive motifs. Furthermore, the strong up-regulation of tested *BjNCED* genes (e.g., *BjNCED1*, *BjNCED4*, *BjNCED7* and *BjNCED16*) under low-temperature condition is consistent with research showing that ABA plays a crucial role in cold tolerance [25]. These findings reinforce the idea that *BjNCED* genes are central to stress tolerance in *B. juncea* and may function similarly to their counterparts in other species.

In summary, this study systematically characterizes the *BjNCED* gene family and identifies that stress-responsive candidate genes in *B. juncea*. *BjNCED1*, *BjNCED4*, *BjNCED7*, *BjNCED16*, and *BjNCED18* displayed striking transcriptional responses to abiotic stimuli. It should be noted that, while this study establishes the genomic organization and transcriptional responses of the *BjNCED* family, we acknowledge that functional validation—such as heterologous overexpression in *A. thaliana* or tobacco, VIGS-mediated silencing in *B. juncea*, or CRISPR/Cas9 knockout—is required to definitively establish the roles of individual *BjNCED* genes in ABA-mediated stress tolerance. Further experiments, such as gene overexpression, gene editing, mutant analysis, and physiological characterization, are essential to validate their functions, which will help explore their potential value for future stress-tolerance breeding programs.

## 4. Materials and Methods

### 4.1. Plant Materials and Stress Treatment

*B. juncea* variety ‘Purple Leaf Mustard’ seeds were obtained from Hunan University of Science and Technology, School of Life and Health Sciences, and the seedlings were grown under a 16 h light/8 h dark photoperiod in an illumination incubator, with a light intensity of 300 µmol·m^−2^·s^−1^, relative humidity was set at 70% and temperature at 25 °C.

Upon attainment of the five-leaf stage, Purple Leaf Mustard plants were subjected to three stress treatments: salt stress (NaCl), low-temperature stress, and exogenous ABA application. All treatments were conducted in three biological replicates. Each biological replicate consisted of approximately 15 seedlings grown in a single 33 cm × 24 cm seedling tray. At each sampling time point (0, 6, 12, 24 and 48 h), leaves from three individual seedlings were collected and pooled to generate one biological replicate sample for RNA extraction. Salt stress was applied by a single irrigation using a 150 mM NaCl solution, and low-temperature stress was applied by transferring plants to a 4 °C growth chamber. For ABA treatment, 0.1 mM ABA was sprayed uniformly onto the leaves. Plant tissues were harvested at 0, 6, 12, 24 and 48 h after the initiation of each treatment. Tender leaves were harvested from treated plants for subsequent experimentation. The collected leaf samples were promptly snap-frozen in liquid nitrogen to maintain RNA integrity and then stored at −80 °C until the extraction of total RNA [33].

### 4.2. Data Sources

The protein sequences of NCED family members in *A. thaliana* were downloaded from the NCBI database (https://ncbi.nlm.nih.gov/?hl=ru) (accessed on 4 February 2026) [47]. The *B. juncea* genome data, including genome sequences, corresponding protein sequences, and the GFF3 annotation file, were sourced from the Ensembl Plants database (https://plants.ensembl.org/index.html) (accessed on 4 February 2026) [48] and searches of Pfam (PF03055) through *A. thaliana* and *B. napus* NCED proteins from the InterPro database (http://www.ebi.ac.uk/interpro/) (accessed on 4 February 2026) [49]. We downloaded the expression data from the *B. juncea* transcriptome database (https://yanglab.hzau.edu.cn/BjuIR) for subsequent analysis (accessed on 6 February 2026) [50].

### 4.3. Identification of the BjNCED Family in B. juncea

Using TBtools software (version 2.142) with default parameters, we performed Blastp and HMM (PF03055) searches, and subsequently discarded duplicate hits and those with E-values greater than 1 × 10^−5^ (https://apps.microsoft.com/detail/9nvr9lb426p2?hl=zh-cn&gl=CN) (accessed on 6 February 2026) [51,52]. A Blastp search was performed against the complete *B. juncea* protein sequences using AtNCED proteins as queries, with a minimum sequence homology threshold of 20%. The overlapping sequences between the HMM search and BLASTP results were considered as candidate *BjNCED* genes. Subsequently, the protein sequences of these candidates were submitted to the Pfam and Conserved Domains Database (CDD) (https://www.ncbi.nlm.nih.gov/Structure/cdd/cdd.shtml) (accessed on 6 February 2026) to further validate the domain integrity. The protein sequences were submitted to the Simple Modular Architecture Research Tool (SMART) (http://smart.embl-heidelberg.de) (accessed on 6 February 2026), which confirmed that 26 out of the 31 candidate BjNCED proteins contain the NCED domain (Table S6)

### 4.4. Analysis of Physicochemical Properties and Subcellular Localization

Using the BjNCED protein sequences, we analyzed their physicochemical properties through the Expasy platform (https://www.expasy.org/) (accessed on 8 February 2026) [53]. The subcellular localization of the BjNCED proteins was predicted using WoLF PSORT (https://wolfpsort.hgc.jp/) (accessed on 8 February 2026) [54].

### 4.5. Phylogenetic Tree Construction

Multiple sequence alignment of NCED protein sequences from *B. juncea*, *A. thaliana*, and *B. napus* was performed using the ClustalW algorithm within MEGA software (version 12.1.2) (https://www.megasoftware.net) (accessed on 12 February 2026) [55,56]. Phylogenetic analysis was performed using the maximum likelihood method implemented in MEGA software, with bootstrap values calculated from 1000 replicates. The optimal amino acid substitution model was LG, with gamma-distributed rate heterogeneity (+G) and a proportion of invariant sites (+I) (Table S5) [52,55]. The final phylogenetic tree was subsequently rendered and optimized for presentation using the EvolView online platform (https://www.evolgenius.info/) (accessed on 12 February 2026) [57].

### 4.6. Gene Structures, Chromosome Localization, and Collinearity Analysis

The exon–intron organization and UTR distribution of the *BjNCED* genes were analyzed using TBtools, based on the *B. juncea* Gene IDs and the GFF3 [51]. Chromosomal locations of *BjNCED* genes were determined from the GFF3 file using Perl scripts and visualized with TBtools [51], based on the retrieved genomic information of *B. juncea* genome from the Ensembl Plants database for collinearity analysis (https://plants.ensembl.org/index.html) (accessed on 12 February 2026) [48]. The duplication events (including tandem and segmental duplications) within the *BjNCED* gene family were identified using TBtools module One Step MCScanX, and parameters were set to default [51].

### 4.7. Cis-Regulatory Element Analysis of BjNCED Promoters in B. juncea

The 2000 bp promoter regions of all *BjNCED* genes were obtained from the *B. juncea* genome, and their cis-elements were predicted via PlantCARE (http://bioinformatics.psb.ugent.be/webtools/plantcare/html/) (accessed on 12 February 2026) [58]. Cis-elements were compiled in Excel 2010, using TBtools to visualize selected *BjNCED* promoters [51].

### 4.8. Phenotype Analysis, Expression Analysis, and qRT-PCR Analysis

To explore stress-induced phenotypic alterations, phenotypic images of *B. juncea* seedlings under salt, ABA, and cold treatments were collected at 0 h and 48 h for comparative analysis.

To investigate the tissue-specific expression profiles of *BjNCED* genes, transcriptome data derived from different *B. juncea* tissues, including silique, leaf, stem, embryo, flower, seed, seed coat, root and flower bud, were imported into TBtools, and then used to generate expression heatmaps [51]. Based on gene structure, phylogenetic analysis, and expression profiles, five representative *BjNCED* genes (Group II: *BjNCED18*; Group V: *BjNCED16*; Group VI: *BjNCED1*, *BjNCED4*, and *BjNCED7*) were finally adopted for qRT-PCR to detect their transcriptional levels under salt stress, ABA treatment, and cold stress. Two genes (Group I: *BjNCED13* and Group II: *BjNCED23*) were omitted from the pilot experiment because their Ct values consistently exceeded 35.

RNA extraction was conducted using AG RNAex Pro Reagent (Accurate Biotechnology Inc., Changsha, China), and the extracted RNA was reverse-transcribed into cDNA using the Evo M-MLV RT Mix Kit with gDNA Clean (Accurate Biotechnology Inc., Changsha, China) with an RNA addition amount of 4 μL. We designed specific qRT-PCR primers using the PrimerQuest website (https://sg.idtdna.com/pages/tools/primerquest) (accessed on 15 February 2026) [59]. For the analysis, the BIOER FQD-96C Real-Time System (Bioer Technology Inc., Hangzhou, China) was utilized with a reaction system of 20 µL. Actin was utilized as the internal reference gene, and the specific gene primers are listed in Table S2. The PCR amplification conditions included 40 cycles of the following steps: pre-denaturation at 95 °C for 20 s, denaturation at 95 °C for 15 s, and annealing at 60 °C for 20 s. An additional melting step was included.

The statistical tests were based on ΔCt values. The 2^−ΔΔCt^ method was employed to calculate relative expression levels, and statistical significance was determined by one-way ANOVA combined with Dunnett’s multiple comparison test using GraphPad Prism software (version 11.0.0) (https://www.graphpad.com/) (accessed on 15 February 2026); meanwhile, assumptions of normality and variance homogeneity were examined, with three technical replicates and biological replicates [60].

## 5. Conclusions

The 26 *BjNCED* genes were not uniformly distributed and were mapped to 15 different chromosomes of the *B. juncea* genome, driven primarily by segmental duplication during family expansion. The promoter regions of these genes harbor cis-acting elements putatively associated with phytohormones, light, and abiotic stress responses. The identified *BjNCED* candidates and their stress-associated expression patterns provide a foundation for future functional studies of ABA biosynthesis and abiotic stress responses in *B. juncea*. Furthermore, qRT-PCR analysis revealed divergent expression patterns of representative *BjNCED* genes under salt, ABA, and low-temperature stresses, with most members exhibiting rapid down-regulation under salt stress and significant induction by cold, highlighting their potential roles in abiotic stress adaptation.

## Figures and Tables

**Table 1:** Physicochemical properties and subcellular localization of BjNCED proteins.

Gene Name	Gene ID	Protein Length (aa)	MW (Da)	pI	Instability Index	Aliphatic Index	GRAVY	Subcellular Localization
*BjNCED1*	*BjuA05g30200S*	597	65,760.44	5.81	40.83	79.38	−0.287	Mitochondrion
*BjNCED2*	*BjuB05g47550S*	580	64,611.82	5.61	42.89	88.90	−0.184	Chloroplast
*BjNCED3*	*BjuB03g11490S*	616	68,128.28	6.56	45.80	80.34	−0.312	Nucleus
*BjNCED4*	*BjuA03g25100S*	592	65,445.10	5.62	43.70	79.70	−0.300	Chloroplast
*BjNCED5*	*BjuA07g07380S*	604	67,210.70	6.03	35.84	83.38	−0.312	Chloroplast
*BjNCED6*	*BjuA09g28340S*	564	62,969.31	5.39	40.97	80.37	−0.326	Chloroplast
*BjNCED7*	*BjuB07g22840S*	589	65,098.86	5.69	43.79	80.61	−0.259	Chloroplast
*BjNCED8*	*BjuB04g17110S*	589	65,376.96	5.61	45.72	79.46	−0.32	Chloroplast
*BjNCED9*	*BjuA09g11990S*	571	64,891.47	6.20	39.59	81.77	−0.279	Chloroplast
*BjNCED10*	*BjuB03g39910S*	589	64,772.73	5.91	45.65	85.06	−0.175	Chloroplast
*BjNCED11*	*BjuA01g35460S*	581	64,419.39	5.32	42.22	85.89	−0.205	Chloroplast
*BjNCED12*	*BjuA09g12000S*	580	66,101.66	6.38	43.70	82.48	−0.363	Chloroplast
*BjNCED13*	*BjuA04g00040S*	534	60,388.41	5.68	35.41	82.64	−0.256	Cytosol
*BjNCED14*	*BjuA07g36950S*	602	66,754.58	5.9	49.15	80.76	−0.299	Chloroplast
*BjNCED15*	*BjuB05g01650S*	591	65,949.05	6.84	39.36	81.59	−0.303	Chloroplast
*BjNCED16*	*BjuB02g60900S*	584	65,025.04	5.34	45.87	86.97	−0.237	Chloroplast
*BjNCED17*	*BjuA02g19730S*	314	34,587.57	9.44	34.26	81.37	−0.258	Chloroplast
*BjNCED18*	*BjuB05g48290S*	594	65,613.83	6.59	49.10	79.92	−0.248	Chloroplast
*BjNCED19*	*BjuA03g14320S*	582	64,724.03	5.55	44.35	86.92	−0.203	Chloroplast
*BjNCED20*	*BjuB06g06720S*	881	98,201.30	6.91	34.27	88.29	−0.169	Chloroplast
*BjNCED21*	*BjuB01g18810S*	586	65,273.51	5.81	33.85	82.94	−0.311	Cytosol
*BjNCED22*	*BjuB07g45570S*	656	72,617.39	6.11	42.08	79.50	−0.287	Chloroplast
*BjNCED23*	*BjuA01g34710S*	596	65,718.88	6.33	46.11	79.98	−0.235	Chloroplast
*BjNCED24*	*BjuA08g12120S*	593	65,437.60	6.45	43.48	84.13	−0.183	Chloroplast
*BjNCED25*	*BjuB01g08830S*	597	65,851.46	5.91	44.81	78.89	−0.303	Mitochondrion
*BjNCED26*	*BjuA01g06830S*	598	65,745.31	5.51	41.64	79.06	−0.273	Chloroplast

## Data Availability

The original contributions presented in this study are included in the article/Supplementary Materials. Further inquiries can be directed to the corresponding author.

## Supplementary Materials

The following supporting information can be downloaded at: https://www.mdpi.com/article/10.3390/plants15152372/s1, Figure S1: NCED unique conserved sequences and conserved histidine sites of 26 members of the *BjNCED* gene family. Table S1: *BjNCED* genes showing 36 gene pairs with segmental duplication. Table S2: Primer sequences for *BjNCED* expression analysis. Table S3: Expression data of the *BjNCED* family from qRT-PCR under salt, ABA, and cold treatments. Table S4: E-value and domain coverage of 26 members of the *BjNCED* gene family. Table S5: The optimal amino acid substitution model. Table S6: Alignment of NCED protein sequences from *B. juncea, B. napus*, and *A. thaliana.*
