# Supplementary material for: Genome-Wide Identification of BjNCED Gene Family and Expression Analysis in Response to ABA, Salt, and Low-Temperature Stresses in Brassica juncea
Source: Plants (Basel). 2026 Aug 1;15(15):2372. doi: 10.3390/plants15152372 (PMC13467608; doi:10.3390/plants15152372)
Supplement: Supplementary file 1 [file plants-15-02372-s001.zip › Figure S1 NCED unique conserved sequence and conserved histidine site of 26 BjNCED gene family.pdf]

1.

***E*-value:** 3.4e-880 [?]   **Site Count:** 26 [?]

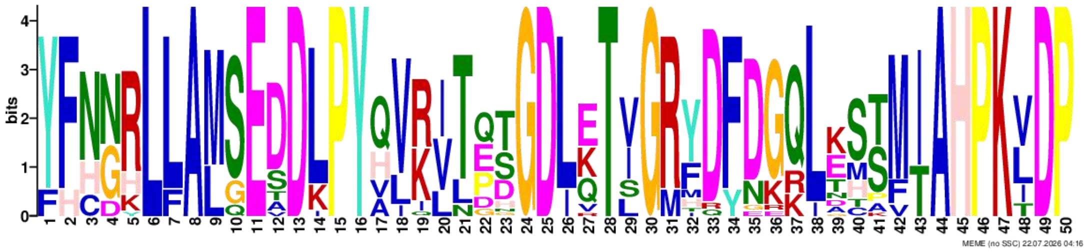

Name [?]	Start [?]	*p*-value [?]	Sites [?]
11. BjNCED11	235	4.71e-60	GVGVANAGLV YFNNRLLAMSEDDLPYQLKITQTGDLETVGRYDFDGQLKSTMIAHPKLDP VTKELHALSY
19. BjNCED19	234	6.13e-60	GVGVANAGLV YFNNRLLAMSEDDLPYQLKITQTGDLQTVGRYDFDGQLKSSMIAHPKLDP VTKELHALSY
26. BjNCED26	253	7.95e-60	GTGVANAGLV YFNNRLLAMSEDDLPYQVRITPSGDLKTIGRYDFDGQLESTMIAHPKVDP ESGELFALSY
25. BjNCED25	253	7.95e-60	GTGVANAGLV YFNNRLLAMSEDDLPYQVRITPSGDLKTVGRYDFDGQLESTMIAHPKVDP ESGELFALSY
22. BjNCED22	314	7.95e-60	GTGVANAGLV YFNNRLLAMSEDDLPYQVRITPSGDLKTVGRYDFDGQLESTMIAHPKVDP ESGELFALSY
16. BjNCED16	234	1.65e-58	GVGVANAGLV YFNNRLLAMSEDDLPYQLKLTQTGDLQTVGRYDFDGQLKSSMIAHPKLDP VSKELHALSY
1. BjNCED1	253	4.65e-58	GTGVANAGLV YFNNRLLAMSEDDLPYQVRITPGGDLKTVGRYDFDGQLESTMIAHPKVDP ESGELFALSY
4. BjNCED4	246	1.25e-57	GTGVANAGLV YFNNRLLAMSEDDLPYQVRITPSGDLKTVGRYDFNGQLESTMIAHPKVDP ESGELFALSY
3. BjNCED3	270	1.82e-57	GLGVANAGLV YFNGHLLAMSEDDLPYHVRVTQTGDLETSGRYDFDGQLKSTMIAHPKLDP ETRELFALSY
14. BjNCED14	256	2.65e-57	GLGVANAGLV YFNGHLLAMSEDDLPYHVKVTQTGDLETSGRYDFDGQLKSTMIAHPKLDP ETRELFALSY
2. BjNCED2	234	1.75e-55	GVGVANAGLV YFNNRLLAMSEDDIPYQLRITQTGDLETVGRYDFEGQLKSTMIAHPKLDP VTKELHALSY
7. BjNCED7	246	5.78e-55	GTGVANAGLV YFNNRLLAMSEVDLPYQVQVTPSGDLKTVGRYDFDGQLESTMIAHPKIDP ESGELFALSY
6. BjNCED6	218	9.31e-54	GTGVANAGLV YFHDRLLAMSEDDLPYQVRVTDDGDLETVGRFDFDGQLNSAMIAHPKIDP ETKELFALSY
17. BjNCED17	241	3.52e-52	GLGVANTGLI YFNGYLLAMSEDDLPYHVIITQTGDLRTSGRYDFNGQLKTTMIAHPKLDP ETGELFALSY
15. BjNCED15	245	2.26e-51	GLGVANTGLI YFNGHLLAMSEDDLPYHLIVTQTGDLKTSGRYDFNGQLKTKMIAHPKLDP ETRELFALSY
8. BjNCED8	243	2.07e-50	GTGVANAGLV YFHDRLLAMSEDDLPYQVRVTDNGDLETVGRFDFNRQLNSAMIAHPKIDP ETKELFALSY
24. BjNCED24	241	2.59e-48	GIGLANTSLA FFCNRLFALGESDLPYAVRLTDTGDIETIGRFDFDGKLEMSMTAHPKTDP QTGETFAFRY
21. BjNCED21	243	6.85e-48	GMGVANAGVV YFNGRLLAMSEDDLPYHVKINGHGDLETIGRFRFDGQIDCPVIAHPKVDP TTGDLHTLSY
23. BjNCED23	247	7.98e-47	GIGLANTSLA FFCNRLFALGESDLPYAVRLTESGDIVTIGRHDFDGKLAMSMTAHPKTDP ETGETFAFRY
5. BjNCED5	261	9.60e-47	GMGVANAGVV YFNGRLLAMSEDDLPYHVKINGHGDLETIGRFQFDEQIDCPVIAHPKVDP TTGDLHTLSY
18. BjNCED18	245	1.05e-46	GIGLANTSLA FFCNRLFALGESDLPYAVRLTESGDIVTIGRHDFDGKLGMSMTAHPKIDP ETGETFAFRY
10. BjNCED10	240	8.46e-46	GIGLANTSLA FFCDRLFALGESDLPYAVRLTETGDIETIGRFDFGGKLAMSMTAHPKTDP QTGETFAFRY
20. BjNCED20	502	1.50e-44	GHGTANTALV YHHGKLLALSEADKPYVVKVLEDGDLQTLGMMDYDKRLTHSFTAHPKVDP TTGEMFTFGY
13. BjNCED13	174	1.50e-44	GHGTANTALV YHHGKLLALSEADKPYVVKVLEDGDLQTLGMMDYDKRLTHSFTAHPKVDP TTGEMFTFGY
12. BjNCED12	218	2.82e-43	GNGTANTSLV YHHGHLLALQETDKPYVIKVLEDGDLQTLGMIDYDKRLTHSFTAHPKVDP VTGEMFTFGY
9. BjNCED9	245	4.99e-43	GNGTANTSLI YHHGKLLALQETDKPYVIKVLEDGDLQTLGMTDYDKRLTHSFTAHPKVDP VTGEMFTFGC

2.

***E*-value:** 3.1e-478 **Site Count:** 25

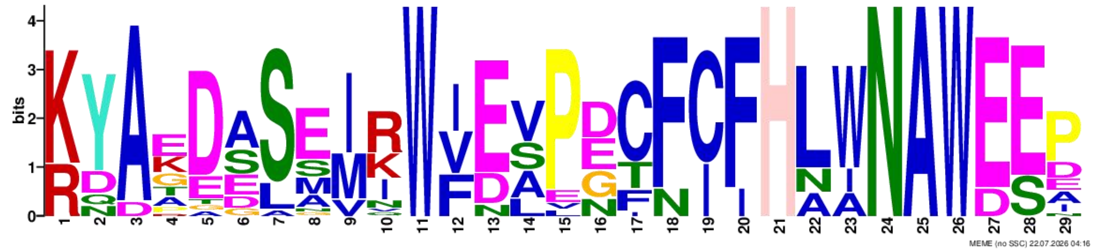

Name	Start	*p*-value		Sites	
4. BjNCED4	384	4.15e-37	EKVARFGVLD	KYAGDSSEIKWIEAPECFCFHLWNAWEEP	ETEEVVVIGS
7. BjNCED7	384	8.61e-37	DKVARFGVLD	KYAEDSSAIRWIEAPECFCFHLWNAWEEP	ETEEIVVIGS
25. BjNCED25	391	3.31e-36	EKVARFGILD	KYAADSSEMRWIEAPDCFCFHLWNAWEEA	ETEEVVVIGS
22. BjNCED22	452	4.09e-36	NKVARFGVLD	KYAEDSSAIKWIEAPGCFCFHLWNAWEEP	ETDEVVVIGS
26. BjNCED26	391	1.37e-35	NKVARFGVLD	KYAGDSSAIKWIEAPGCFCFHLWNAWEEP	ETEEVVVIGS
1. BjNCED1	391	2.01e-35	EKVARFGILD	KYAADSSGIRWIEAPDCFCFHLWNAWEEP	ETEEVVVIGS
14. BjNCED14	394	1.71e-34	NKKSRFGVLD	KYAEDASSMRWIEVEDCFCFHLWNAWEEA	ETDEVVVIGS
3. BjNCED3	408	1.71e-34	NKKSRFGVLN	KYAKDASSMRWIEVEDCFCFHLWNAWEEA	ETDEVVVIGS
6. BjNCED6	356	3.95e-33	EKVSRFGILP	KNAEDASEMIWVESPDTFCFHLWNAWESP	ETDEVVVIGS
8. BjNCED8	381	5.28e-33	EKVSRFGILP	KNAEDASEIIWVESPETFCFHLWNAWESP	ETDEVVVIGS
15. BjNCED15	383	8.34e-32	KKRSRFGVLN	KYARDAASIRWIDVVDCFCFHLWNAWEEP	DTDEVVVIGS
2. BjNCED2	372	7.24e-31	DKVSRLGIMP	KDATEASEIIWVDSPETFCFHLWNAWESP	ETEEVVVIGS
20. BjNCED20	640	2.15e-30	TKKARFGVLP	RYAKDELMIRWFELPNCFIFHNANAWEEE	DEVVLITCRL
13. BjNCED13	312	2.15e-30	TKKARFGVLP	RYAKDELMIRWFELPNCFIFHNANAWEEE	DEVVLITCRL
12. BjNCED12	356	2.73e-30	TKKARFGVLP	RYAKDELMIKWFELPNCFIFHNANAWEED	EEVVLITCRL
9. BjNCED9	383	3.46e-30	TKNARFGVLP	RYAKDDLMIRWFELPNCFIFHNANAWEEE	DEVVLITCRL
11. BjNCED11	373	6.20e-30	GKVSRLGIMP	KDATEASEIVWVDSPETFCFHLWNAWESP	ETEEVVVIGS
16. BjNCED16	372	1.23e-29	EKVSRLGIMP	KDAAEASEVIWVNSPETFCFHLWNAWESP	ETEEVVVIGS
19. BjNCED19	372	3.79e-29	EKVSRLGIMP	KDATEASEVIWVNSPEIFCFHLWNAWESP	ETEEVVVIGS
18. BjNCED18	383	3.28e-28	GKTPRLGVIP	RYAEDESEMRWFEVPGFNIIHAINAWDED	DGNTIVLIAP
21. BjNCED21	381	3.64e-28	EKMSRFGVLS	KQDPAGSNINWVDVPDCFCFHLWNAWEEI	TEDGDLVIVV
5. BjNCED5	399	1.03e-27	EKMSRFGVLS	KQDPTGSSVNWVDVPDCFCFHLWNAWEEI	AEDGDPVIVV
10. BjNCED10	381	1.40e-27	RKTPRLGVIP	RYAGDDSEMKWFEVPGFNIIHAINAWDED	DGNAVVLIAP
23. BjNCED23	385	2.04e-26	GKTPRLGVIP	RYAEGESEMKWFEVPGFNIIHAINAWDED	DGDTIVLIAP
24. BjNCED24	382	1.64e-25	RKTPRLGVIP	RYAGDDSEMQWFEVLGFNIIHAINAWDEN	DGTTVVLIAP

3.

***E*-value:** 2.8e-515 ?   **Site Count:** 25 ?

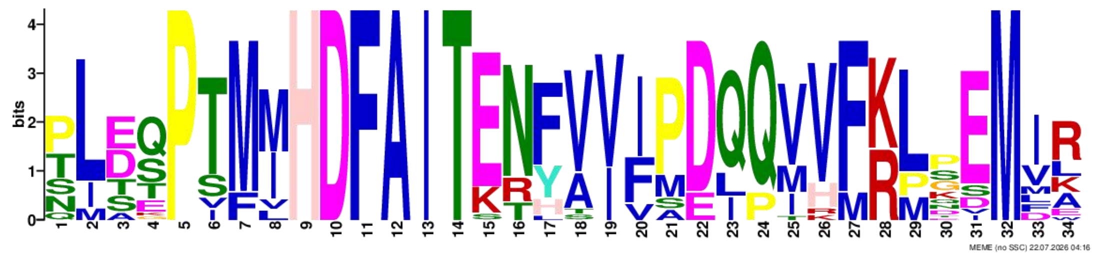

Name ?	Start ?	*p*-value ?	Sites ?		
26. BjNCED26	338	2.44e-43	DGTKSPDVEI	NLEQPTMMHDFAITENFVVIPDQQVVFKLPEMIR	GGSPVVYDEN
22. BjNCED22	399	2.44e-43	DGTKSPDVEI	NLEQPTMMHDFAITENFVVIPDQQVVFKLPEMIR	GGSPVVYDEN
7. BjNCED7	331	4.33e-43	DGIKSPDVEI	NLDQPTMMHDFAITENFVVIPDQQVVFKLPEMIR	GGSPVVYDKD
4. BjNCED4	331	4.33e-43	DGIKSPDVEI	NLDQPTMMHDFAITENFVVIPDQQVVFKLPEMIR	GGSPVVYDKE
1. BjNCED1	338	1.01e-42	DGEKSPDVEI	QLDQPTMMHDFAITENFVVIPDQQVVFKLQEMIR	GGSPVIYDKE
5. BjNCED5	346	2.41e-41	CGTKTRDLDI	TLEQPTMIHDFAITENFVVIPDQQMVFKLSEMIR	GGSPVIYDRE
25. BjNCED25	338	1.67e-40	DGTKSPDVQI	QLDQPTMMHDFAITENFVVVPDQQVVFKLQEMIR	GGSPVIYDKE
14. BjNCED14	341	7.41e-40	DGQKSPDVEI	PLDQPTMIHDFAITENFVVVPDQQVVFRLPEMIR	GGSPVVYDKN
21. BjNCED21	328	1.35e-39	CGKKTRDVDI	TLEQPTMIHDFAITKNFVVIPDQQMVFKLSEMIW	GGSPVIYDRE
19. BjNCED19	319	4.98e-39	DGVKSPELEI	PLETPTMIHDFAITENFVVIPDQQVVFKLGEMIA	GNSPVVFDGE
16. BjNCED16	319	4.98e-39	DGVKSPELEI	PLETPTMIHDFAITENFVVIPDQQVVFKLGEMIA	GNSPVVFDGE
11. BjNCED11	320	1.79e-37	DGVKSPEVEI	PLETPTMIHDFAITENFVVIPDQQVVFKLGEMMA	GNSPVVFDGG
3. BjNCED3	355	1.79e-37	DGQKSPDVEI	PLDQPTMIHDFAITENLVVVPDQQVVFRLPEMIR	GGSPVVYDKN
2. BjNCED2	319	1.79e-37	DGVKSPEVEI	PLETPTMIHDFAITENFVVIPDQQVVFKLGEMMA	GNSPVVFDGD
15. BjNCED15	330	2.25e-36	NGEKSPDVEI	PLDGPTMIHDFAITENYVVIPDQQVVFRLTEMVR	GGSPVVYDKK
8. BjNCED8	328	3.22e-35	EGEKSPDVEI	PLASPTMMHDFAITENFVVIPDQQVVFKLSDMFL	GKSPVKYDGE
6. BjNCED6	303	3.22e-35	EGEKSPDVEI	PLASPTMMHDFAITENFVVIPDQQVVFKLSDMFL	GKSPVKYDGE
13. BjNCED13	258	6.60e-30	DGFMHDPVPI	TISEPIMMHDFAITETYAIFMDLPMHFRPKEMVK	EKKMVYSFDP
20. BjNCED20	586	9.37e-30	DGIMHDPVPI	TISEPVMMHDFAITETYAIFMDLPMHFRPKEMVK	EKKMVYSFDP
12. BjNCED12	302	3.70e-29	DGIMHDPVPI	TISEPIMMHDFAITENYSIFMDLPTHFRPKEMVK	EKKMLYSFDP
9. BjNCED9	329	4.03e-29	DGIMHDPVPI	TISKPVMMHDFAITETYAIFMDLPMHFRPEEMVK	EKKMIYSFDP
10. BjNCED10	325	7.22e-26	GKKQRDVPVY	SLTSPSFVHDFAITKRHAIFAEIQIVMRMNVMDL	VLEGGSPVAA
24. BjNCED24	326	1.74e-25	GKKQRDVPVY	SLTSPSFVHDFAITKRHTIFAEIQIVMRMNIMDL	VLEGGSPVGA
18. BjNCED18	330	4.78e-25	GKKQKDVPVF	SMTSPSFLHDFAITKRHAIFSEIQIKMRMDSMLE	GGSPVGADNG
23. BjNCED23	332	1.05e-24	GNKRKDVPVF	SMTSPSFLHDFAITSRYAIFSEIQIRMRMDSMLE	GGSPVGADNG

4.

***E*-value:** 9.4e-408 **Site Count:** 22

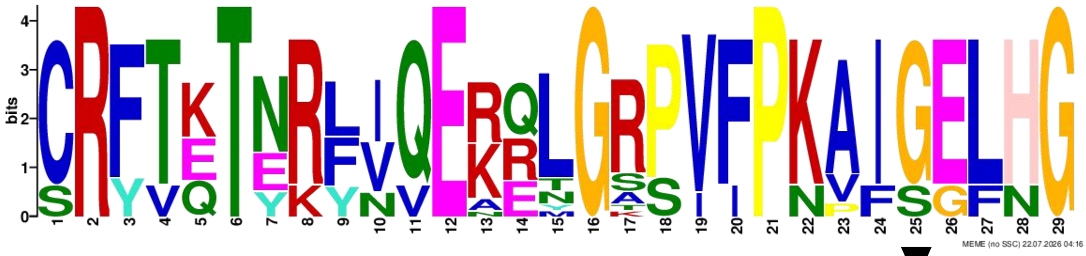

25.	BjNCED25	191	6.70e-38	KFEDGSASYA	CRFTQTNRFIQERQLGRPVFPKAIGELHG HTGIARLMLF
22.	BjNCED22	252	6.70e-38	KFEDGSASYA	CRFTQTNRFIQERQLGRPVFPKAIGELHG HTGIARLMLF
4.	BjNCED4	184	6.70e-38	KFEDGSASYA	CRFTQTNRFIQERQLGRPVFPKAIGELHG HTGIARLMLF
1.	BjNCED1	191	6.70e-38	KFEDGSASYA	CRFTQTNRFIQERQLGRPVFPKAIGELHG HTGIARLMLF
26.	BjNCED26	191	5.70e-35	KFEDGSASYA	CRFTQTNRFIQERQLGKPVFPKAIGELHG HTGIARLMLF
7.	BjNCED7	184	6.70e-35	KFEDGLASYA	CRFTQTNRFIQERRMGRPVFPKAIGELHG HTGIARLMLF
19.	BjNCED19	172	9.49e-35	KITNGSASYA	CRFTKTERLIQEKRLGRPVFPKAIGELHG HSGIARLMLF
11.	BjNCED11	173	9.49e-35	KITNGSASYA	CRFTKTERLIQEKRLGRPVFPKAIGELHG HSGIARLMLF
2.	BjNCED2	172	9.49e-35	KITNGSASYA	CRFTKTERLIQEKRLGRPVFPKAIGELHG HSGIARLMLF
16.	BjNCED16	172	1.80e-34	KISNGSASYA	CRFTETERLIQEKRLGRPVFPKAIGELHG HSGIARLMLF
3.	BjNCED3	208	3.92e-34	RFNNGSVSYS	CRFTETNRYVQERELGRSVFPKAIGELHG HLGIAKLMLF
14.	BjNCED14	194	4.55e-34	RFNNGSVSYS	CRFTETNRFVQERELGRSVFPKAIGELHG HLGIAKLMLF
17.	BjNCED17	179	5.26e-33	RFNNGSVSYS	CRFTETNRLVQERELGRSIFPKAIGELHG HLGIAKLMLF
15.	BjNCED15	183	1.25e-32	RFNNGSVSYS	CRFTETNRFVQEREYGRSVFPKAIGELHG HLGIAKLMLF
21.	BjNCED21	181	4.62e-32	IGSDNRVSYS	CRYTKTNRLVQEAELGRPVFPKPIGELHG HSGLARLALF
8.	BjNCED8	181	5.81e-32	KISDGDASYS	CRFTETERLVQEKRLGSPIFPKAIGELHG HSGIARLMLF
6.	BjNCED6	156	5.81e-32	KITNGDASYS	CRFTETERLVQEKRLGSPIFPKAIGELHG HTGIARLMLF
5.	BjNCED5	199	2.23e-31	IGSDNQVSYS	CRYTKTNRLVQEAELGRSVFPKPIGELHG HSGLARLALF
24.	BjNCED24	178	2.57e-23	RIRNGKATLC	SRFVKTYKYNVEKQNGAPVIPNVFSGFNG VTASVARGAL
18.	BjNCED18	182	3.69e-23	RIRDGKATLC	SRYVKTYKYNVEKQTGSPVIPNVFSGFNG VPASMARGAL
23.	BjNCED23	184	6.12e-23	RIRDGKATLC	SRYVKTYKYNVEKQTGTPVIPNVFSGFNG VPASMARGAL
10.	BjNCED10	177	1.78e-22	RIRNGKATLC	SRYVKTYKYNVENQNGAPVIPNVFSGFNG VTASLARGAL

5.

***E*-value:** 2.4e-582 **Site Count:** 23

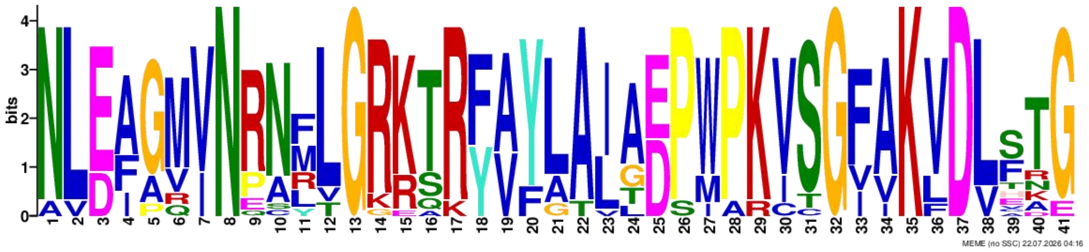

6. BjNCED6	439	4.78e-51	RTIIPGSVQM	NLEAGMVNRNFLGRKTRYAYLAIAEPWPKVSGFAKVDLSTG	EVKNHFYGDK
26. BjNCED26	475	2.91e-50	PIISDGEEQV	NLEAGMVNRNMLGRKTRYAYLALAEPWPKVSGFAKVDLFTG	EVEKHLYGDD
7. BjNCED7	465	6.24e-50	TRRPIISDGV	NLEAGMVNRNMLGRKTRFAYLALAEPWPKVSGFAKVDLFTG	EVKKYLYGGD
4. BjNCED4	468	6.24e-50	PIISDGEEQV	NLEAGMVNRNMLGRKTRFAYLALAEPWPKVSGFAKVDLFTG	EVKKYLYGGD
8. BjNCED8	464	1.04e-49	RTIIPGSVQM	NLEAGMVNRNLLGRKTRYAYLAIAEPWPKVSGFAKVDLSTG	EVKNHFYGGK
22. BjNCED22	533	1.27e-48	TRRPIIDHQV	NLEAGMVNRNMLGRRTRYAYLALAEPWPKVSGFAKVDLFTG	EVEKHLYGDG
14. BjNCED14	475	3.21e-47	TRRPVISEEL	NLEAGMVNRNLLGRKTRFAYLALTEPWPKVSGFAKVDLSTG	EVQKYIYGDG
3. BjNCED3	489	3.21e-47	TRRPVISEQL	NLEAGMVNRNLLGRKTRFAYLALTEPWPKVSGFAKVDLSTG	EVRKYIYGSG
25. BjNCED25	475	3.82e-47	PIISDGEEQV	NLEAGMVNRNMLGRKTKFAYLALAEPWPKVSGFAKVDLSTG	EVEKHLYGEN
16. BjNCED16	453	7.58e-47	TRRALLDDEV	NLEIGMVNRNRLGRKTRFVFLAIAEPWPKVSGFAKVDLFTG	EIEKYIYGGE
15. BjNCED15	465	4.50e-46	RRPVIISQQL	NLEAGMVNRNLLGRKTRFAYLAITEPWPKVSGFAKVDLSDG	DVQKYIYGDG
1. BjNCED1	475	5.25e-46	PIISDGDQQV	NLEAGMVNRNMLGRKTKFAYLALAEPWPKVSGFAKVDLATG	EVKKHLYGDD
19. BjNCED19	453	7.15e-46	TRRALLDDEV	NLEIGMVNRNRLGRKTRFAFLAIADPWPKVCGFAKVDLCTG	KIEKYIYGGE
2. BjNCED2	453	4.22e-45	TRQALLDEEV	NLEIGMVNRNRLGRKARFAFLAIADPWPKVSGFAKVDLFTG	DIKKYVYGSE
11. BjNCED11	454	8.51e-44	TRRAMLVDEV	NLEIGMVNRNRLGRETRFAFLAIADPWPKVSGFAKVDLVTG	EIEKYVYGDG
21. BjNCED21	464	6.35e-42	SNRKVIVTGM	NLEAGQVNQNFLGRKTRFVYLAIADPWPRCSGIAKVDLENG	TVIEFKYGPG
5. BjNCED5	482	1.28e-41	SNRKVLVTGM	NLEAGQVNRNFLGRKTRFVYLAVADPWPRCSGIAKVDLENG	TVIQFKYGPG
10. BjNCED10	459	4.10e-35	IVTRHPISSR	NLDFAVINPAFLGRRSRYVYAAIGDPMPKISGVVKLDVSKG	DRDDFTVARR
23. BjNCED23	463	8.55e-34	IVTRHPISAR	NLDFAVINPAFVGRRSRYVYAAIGDPMPKISGVVKLDVTRG	DRDDCTVARR
18. BjNCED18	461	8.55e-34	IVTRHPISAR	NLDFAVINPAFVGRRSRYVYAAIGDPMPKISGVVKLDVTRG	DRDDCTVARR
24. BjNCED24	460	2.17e-33	IVTRHPISAR	NLDFAVINPAFLGGRSRYVYAAIGDPMPKISGVVKLDVSKG	DRDDCTVARR
13. BjNCED13	394	4.92e-27	SASQKKLSPS	AVDFPRINECYTGKKQRYVYGTILDSIAKVTGIIKFDLHAE	AETGKEILEV
20. BjNCED20	722	1.60e-26	SASQKKLSAS	AVDFPRINESYTGKKQRYVYGTILDSIAKVTGIIKFDLHAE	AETGKRMLEV

6.

***E*-value:** 9.2e-497 **Site Count:** 25

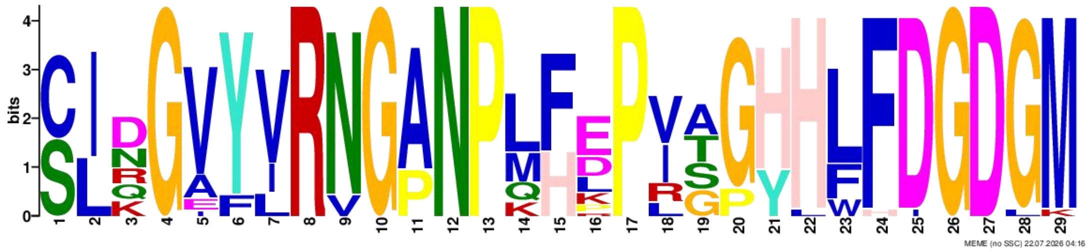

**Name**	**Start**	***p*-value**		**Sites**	
19. BjNCED19	129	6.34e-37	CLDVEGTIPD	CIDGVYVRNGANPMFEPIAGHHLFDGDGM	VHAVKITNGS
16. BjNCED16	129	6.34e-37	CLDVEGTIPD	CIDGVYVRNGANPMFEPIAGHHLFDGDGM	VHAVKISNGS
17. BjNCED17	136	6.40e-35	NLPVTGTLPE	CIQGVYVRNGANPLHKPISGHHLFDGDGM	VHAVRFNNGS
14. BjNCED14	151	6.40e-35	NLPVTGTVPE	CIQGVYVRNGANPLHKPISGHHLFDGDGM	IHAVRFNNGS
3. BjNCED3	165	6.40e-35	NLPVTGTLPE	CIQGVYVRNGANPLHKPISGHHLFDGDGM	VHAVRFNNGS
8. BjNCED8	138	3.49e-34	SLSVTGKIPD	CIDGVYLRNGANPLFEPVSGHHLFDGDGM	VHAVKISDGD
6. BjNCED6	113	3.49e-34	SLSVTGKIPD	CIDGVYLRNGANPLFEPVSGHHLFDGDGM	VHAVKITNGD
15. BjNCED15	140	4.88e-34	NLPVTGTLPE	CIQGVYVRNGANPLHRPISGHHLFDGDGM	VHAIRFNNGS
11. BjNCED11	130	4.88e-34	FLTVEGTIPD	SIDGVYVRNGANPMFDPIAGHHLFDGDGM	VHAIKITNGS
2. BjNCED2	129	4.88e-34	FLPVEGTIPD	SIDGVYVRNGANPMFDPIAGHHLFDGDGM	VHAVKITNGS
26. BjNCED26	148	1.90e-33	NLPVVGTIPD	SIKGVYVRNGANPLHEPVTGHHFFDGDGM	VHAVKFEDGS
25. BjNCED25	148	1.90e-33	NLPVVGKIPD	SIRGVYVRNGANPLHEPVTGHHFFDGDGM	VHAVKFEDGS
22. BjNCED22	209	1.90e-33	NLPVVGKIPD	SIKGVYVRNGANPLHEPVTGHHFFDGDGM	VHAVKFEDGS
7. BjNCED7	141	1.90e-33	NLPVVGKIPD	SIRGVYVRNGANPLHEPVTGHHFFDGDGM	VHAVKFEDGL
4. BjNCED4	141	1.90e-33	NLPVVGKIPD	SIKGVYVRNGANPLHEPVTGHHFFDGDGM	VHAVKFEDGS
1. BjNCED1	148	1.90e-33	NLPVVGKIPD	SIKGVYVRNGANPLHEPVTGHHFFDGDGM	VHAVKFEDGS
21. BjNCED21	137	1.62e-32	GLEVVGQIPP	CLRGVYVRNGANPMFPPLGGHHLFDGDGM	IHAVSIGSDN
5. BjNCED5	155	3.48e-32	GLEVIGNIPP	CLRGVYIRNGANPMFPPLGGHHLFDGDGM	IHAVSIGSDN
24. BjNCED24	135	2.28e-29	CEIIHGSLPP	SLDGAYIRNGPNPQFLPRGPYHLFDGDGM	LHAIRIRNGK
10. BjNCED10	134	2.28e-29	CEIIHGSLPP	SLDGAYIRNGPNPQFLPRGPYHLFDGDGM	LHAIRIRNGK
23. BjNCED23	141	2.72e-29	CQIIHGSLPP	SLNGAYIRNGPNPQFLPRGPYHLFDGDGM	LHAIRIRDGK
18. BjNCED18	139	2.72e-29	CEIIHGSLPP	SLNGAYIRNGPNPQFLPRGPYHLFDGDGM	LHAIRIRDGK
13. BjNCED13	70	3.86e-29	LPVVHGFLPE	CLNGEFVRVGPNPKFDPVAGYHWFDGDGM	IHAVRIKDGK
9. BjNCED9	141	2.51e-28	DLHVHGFLPE	CLNGIFLRVGPNPKFDPLAGYHWFDGDGM	IHGVRIKDGK
12. BjNCED12	155	9.65e-22	DLPVHGFLPE	CLNGEFLRVGPNPKFDPVAGYLWHIGDLK	GIFGLLMANM

7.

***E*-value:** 2.2e-409 **Site Count:** 24

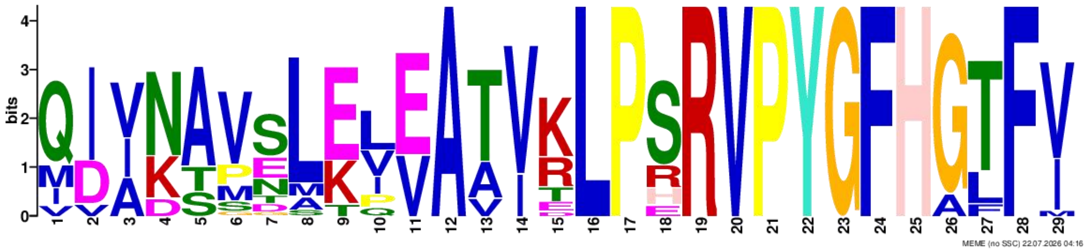

	Name	Start	*p*-value	Sites		
26.	BjNCED26	561	6.42e-35	HDEMTWKSEL	QIVNAVSLEVEATVKLPSRVPYGFHGTFI	GASDLAKQV
25.	BjNCED25	560	6.42e-35	HDEKTWKSEL	QIVNAVSLEVEATVKLPSRVPYGFHGTFI	GASDLAKQV
22.	BjNCED22	619	6.42e-35	HDEMTWKSEL	QIVNAVSLEVEATVKLPSRVPYGFHGTFI	GASDLAKQV
7.	BjNCED7	552	6.42e-35	HDEMTWKSEL	QIVNAVSLEVEATVKLPSRVPYGFHGTFI	GANDLATQE
4.	BjNCED4	555	6.42e-35	HDEETWKSEL	QIVNAVSLEVEATVKLPSRVPYGFHGTFI	GANDLATQV
1.	BjNCED1	560	6.42e-35	HDEMTWTSEL	QIVNAVSLEVEATVKLPSRVPYGFHGTFI	GASDLANQV
2.	BjNCED2	543	1.16e-33	HDEEKETSVL	QIINAVNLKLEATIKLPSRVPYGFHGTFV	DSSELVDQV
8.	BjNCED8	552	3.55e-33	HDEESWKSEL	QIVNAVTLELEATIKLPSRVPYGFHGTFV	NSADMLNQA
6.	BjNCED6	527	3.55e-33	HDEEGWKSEL	QIVNAVTLELEATIKLPSRVPYGFHGTFV	NSADMLNQA
19.	BjNCED19	545	2.09e-32	HDEEKETSEL	QIINAVNLTLEATIKLPSRVPYGFHGTFV	DSSELVDQA
16.	BjNCED16	547	2.09e-32	HDEEKETSEL	QIINAVNLTLEATIKLPSRVPYGFHGTFV	DSSELVDQS
11.	BjNCED11	544	2.05e-31	HDEEKETSEL	QIIDAVDLKLEATIKLPSRVPYGFHGTFV	DASELVDQV
3.	BjNCED3	577	2.75e-31	HDEENVKSEL	QIINAVGLKLEATVTLPSRVPYGFHGTFI	SNEDLLKQAL
14.	BjNCED14	563	4.82e-31	HDEENVRSEL	QIINAGSLKLEATVTLPSRVPYGFHGTFI	SNEDLLKQAL
15.	BjNCED15	552	8.26e-31	HDEEKVKSEL	QIINTVNLKLEATVTLPSRVPYGFHGTFM	SNDDLMNQAL
24.	BjNCED24	557	1.41e-27	EVAGESKFLV	MDAKSPELEIVAAVRLPRRVPYGFHGLFV	KESDVNKL
23.	BjNCED23	560	1.41e-27	EVAGESKFLV	MDAKSPELEIVAAVRLPRRVPYGFHGLFV	KESDLNKL
18.	BjNCED18	558	1.41e-27	EVAGESKFLV	MDAKSPELEIVAAVRLPRRVPYGFHGLFV	KESDLNKL
10.	BjNCED10	556	1.41e-27	EVSGESKFLV	MDAKSPELEIVAAVRLPRRVPYGFHGLFV	KESDI
5.	BjNCED5	567	5.06e-26	RDEERDESEF	VVVDASEMKQVAAVRLPERVPYGFHGTFV	SENQLKEQV
21.	BjNCED21	549	9.00e-26	RDEERDESEF	VVVDASDMKQVAAVRLPERVPYGFHGTFV	SENQLKEQI
13.	BjNCED13	495	7.75e-25	ENTGKSCVNV	IDAKTMSAEPVAVVELPHRVPYGFHAFFV	TEEQLQEQTL
12.	BjNCED12	541	9.77e-25	ENTGKSCVNV	IDAKTMSSEPVAVVELPHRVPYGFHALFV	TEEQLQEQTL
20.	BjNCED20	823	1.23e-24	ENTGKSCVNV	IDAKTMSAEPVAVVDLPHRVPYGFHAFFV	TEREIQMEAL

8.

***E*-value:** 4.4e-364 **Site Count:** 26

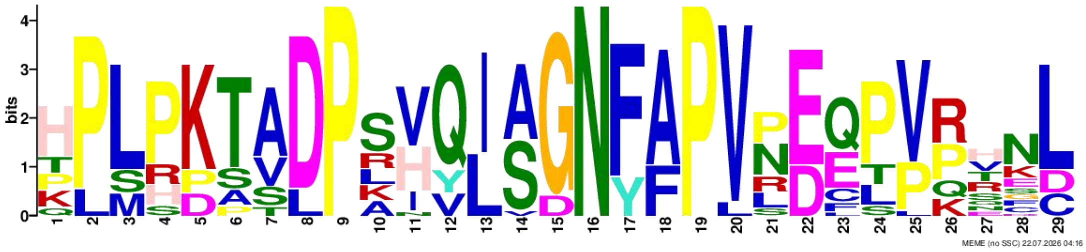

4.	BjNCED4	104	9.72e-36	EGLLVSHERQ	HPLPKTADPSVQIAGNFAPVNEQPVRHNL PVVGKIPDSI
26.	BjNCED26	111	1.33e-34	EGFLVSHERQ	HPLPKTADPSVQIAGNFAPVNEQPVRRNL PVVGTIPDSI
25.	BjNCED25	111	1.33e-34	EGFLVSHERQ	HPLPKTADPSVQIAGNFAPVNEQPVRRNL PVVGKIPDSI
7.	BjNCED7	104	7.04e-34	EGFLVCHERQ	HPLPKTADPSVQIAGNFAPVNEQPVRSNL PVVGKIPDSI
17.	BjNCED17	99	6.26e-33	NAMVSYERRH	HPLPKTADPAVQIAGNFFPVPEQPVQHNL PVTGTLPECI
15.	BjNCED15	103	6.26e-33	NAIVSHERRH	HPLPKTADPAVQIAGNFFPVPEQPVQHNL PVTGTLPECI
14.	BjNCED14	114	1.87e-32	NAMVSHERRR	HPLPKTADPAVQIAGNFFPVPEQPVKHNL PVTGTVPECI
3.	BjNCED3	128	1.87e-32	KAMVSHERRR	HPLPKTADPAVQIAGNFFPVPEQPVKHNL PVTGTLPECI
22.	BjNCED22	172	1.37e-31	EGFLVSHERQ	HPLPKTADPSVQIVGNFAPVNEQPVRRNL PVVGKIPDSI
1.	BjNCED1	111	1.65e-31	EGFLVSHERQ	HPLPKTADPSVQIAGNFAPVNEQPLRRNL PVVGKIPDSI
19.	BjNCED19	92	6.36e-28	ERVLISREQD	TPLPKTADPRIQIAGNYFPVPEFPVRECL DVEGTIPDCI
16.	BjNCED16	92	6.36e-28	ERALISREQD	TPLPKTADPRIQIAGNYFPVPEFPVRECL DVEGTIPDCI
11.	BjNCED11	93	4.04e-27	ERALISHEKD	TPLSKTADPSVQIAGNYFPVSECPVRQFL TVEGTIPDSI
8.	BjNCED8	101	4.52e-27	EAALLSRERS	QPLPKTVDPRHQISGNYAPVPEQSVKSSL SVTGKIPDCI
6.	BjNCED6	76	4.52e-27	EAALLSRERS	QPLPKTVDPRHQISGNYAPVPEQSVKSSL SVTGKIPDCI
21.	BjNCED21	100	5.04e-27	SSLVFPMEQN	HPLPKPTDPSIQLSGNFAPVNECPVQNGL EVVGQIPPCL
5.	BjNCED5	118	5.04e-27	SSLVVPMEQN	HPLPKPTDPSIQLSGNFAPVNECPVQNGL EVIGNIPPCL
2.	BjNCED2	92	1.13e-25	ERALISHEKN	TPLSKTADPSVQIAGNYFPVSELPVRQFL PVEGTIPDSI
24.	BjNCED24	97	5.12e-22	VEDVINTFID	PPSRPSVDPKHVLSGNFAPVLDELPPTEC EIIHGSLPPS
23.	BjNCED23	103	3.10e-21	MEEVINTFID	PPSRPSVDPKHVLSDNFAPVLDELPPTEC QIIHGSLPPS
18.	BjNCED18	101	3.10e-21	MEEVINTFID	PPSRPSVDPKHVLSDNFAPVLDELPPTEC EIIHGSLPPS
20.	BjNCED20	347	3.86e-21	LLDLVERVVV	KLMHDASLPLHYLSGNFAPVRDETPPVKD LPVVHGFLPE
13.	BjNCED13	31	3.86e-21	LLDLVERVVV	KLMHDASLPLHYLSGNFAPVRDETPPVKD LPVVHGFLPE
10.	BjNCED10	96	5.97e-21	VEDIINTFID	PPSRPSVDPKHVLSDNFAPVLDELPPTDC EIIHGSLPPS
9.	BjNCED9	103	2.65e-20	LIDLLEALVV	KLMHDASLPLHYLSGNFAPLRDETPPVKD LHVHGFLPEC
12.	BjNCED12	117	4.31e-20	LIDLLESLLV	KLMHDTSLPLNYLSGNFAPLRDETPPVKD LPVHGFLPEC

9.

***E*-value:** 2.4e-328 **Site Count:** 17

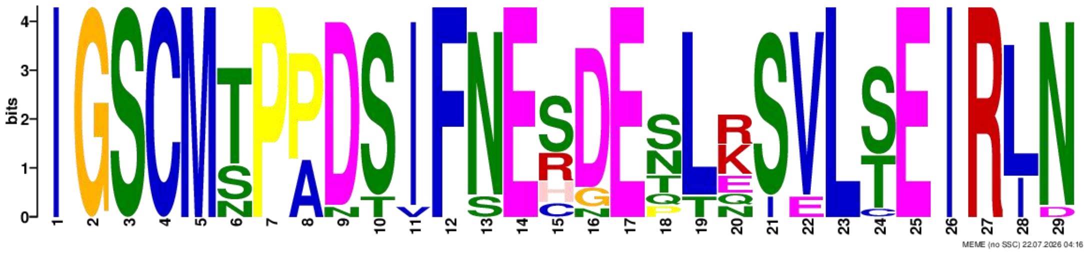

	Name	Start	*p*-value	Sites
26.	BjNCED26	427	2.42e-36	EEPETEEVVV IGSCMTPPDSIFNESDENLKSVLSEIRLN LRTGESTRRP
4.	BjNCED4	420	2.42e-36	EEPETEEVVV IGSCMTPPDSIFNESDENLKSVLSEIRLN LRTGESTRRP
25.	BjNCED25	427	4.23e-36	EEAETEEVVV IGSCMTPPDSIFNESDESLRSVLSEIRLN LRTGESTRRP
22.	BjNCED22	488	7.77e-36	EEPETDEVVV IGSCMTPPDSIFNESDESLQSVLSEIRLN LRTGESTRRP
14.	BjNCED14	430	3.62e-35	EEAETDEVVV IGSCMTPPDSIFNEHDETLQSVLSEIRLN LRTGESTRRP
8.	BjNCED8	417	6.57e-35	ESPETDEVVV IGSCMTPADSIFNECDEQLNSVLSEIRLN LKTGKSTRRT
6.	BjNCED6	392	6.57e-35	ESPETDEVVV IGSCMTPADSIFNECDEQLNSVLSEIRLN LKTGKSTRRT
1.	BjNCED1	427	6.57e-35	EEPETEEVVV IGSCMTPPDSIFNESDENLKSVLCEIRLN LRTGESTRRP
7.	BjNCED7	420	1.09e-33	EEPETEEIVV IGSCMTPPDSIFNESNENLESVLSEIRLN LRTGESTRRP
15.	BjNCED15	419	1.69e-33	EEPDTDEVVV IGSCMTPPDSVFNEHDETLESVLSEIRLN LKTGESTRRP
11.	BjNCED11	409	1.69e-33	ESPETEEVVV IGSCMSPADSIFNERDESLKSVLTEIRIN LRTRESTRRA
2.	BjNCED2	408	1.69e-33	ESPETEEVVV IGSCMSPADSIFNERDESLKSVLTEIRIN LRTRESTRQA
19.	BjNCED19	408	1.88e-33	ESPETEEVVV IGSCMSPADSIFNERDESLRSVLTEIRIN LRTREATRRA
16.	BjNCED16	408	1.88e-33	ESPETEEVVV IGSCMSPADSIFNERDESLRSVLTEIRIN LRTREATRRA
3.	BjNCED3	444	2.98e-33	EEAETDEVVV IGSCMTPPNSIFNEHDETLESVLSEIRLN LKTGESTRRP
5.	BjNCED5	438	2.22e-27	AEDGDPVIVV IGSCMNPPDTIFSESGEPTRIELTEIRLN LRTKESNRKV
21.	BjNCED21	420	5.51e-26	TEDGDLVIVV IGSCMNPPDTIFSESGEPTRIELTEIRLD LRTKESNRKV

10.

***E*-value:** 3.1e-264 **Site Count:** 26

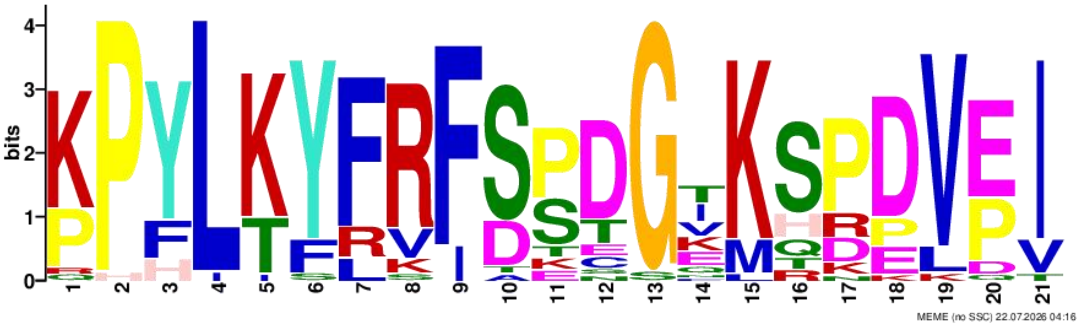

Name	Start	*p*-value	Sites		
7. BjNCED7	310	7.89e-27	LFALSYDVVS	KPYLKYFRFSPDGIKSPDVEI	NLDQPTMMHD
26. BjNCED26	317	1.65e-26	LFALSYDVVS	KPYLKYFRFSPDGTKSPDVEI	NLEQPTMMHD
22. BjNCED22	378	1.65e-26	LFALSYDVVS	KPYLKYFRFSPDGTKSPDVEI	NLEQPTMMHD
1. BjNCED1	317	3.88e-26	LFALSYDVVS	KPYLKYFRFSPDGEKSPDVEI	QLDQPTMMHD
14. BjNCED14	320	1.74e-25	LFALSYDVVS	KPYLKYFRFSSDGQKSPDVEI	PLDQPTMIHD
25. BjNCED25	317	8.89e-25	LFALSYDVVS	KPYLKYFRFSPDGTKSPDVQI	QLDQPTMMHD
11. BjNCED11	299	1.38e-24	LHALSYDVVR	KPYLKYFRFSPDGVKSPEVEI	PLETPTMIHD
2. BjNCED2	298	1.38e-24	LHALSYDVVR	KPYLKYFRFSPDGVKSPEVEI	PLETPTMIHD
8. BjNCED8	307	5.45e-24	LFALSYDVVK	KPYLKYFRFSPEGEKSPDVEI	PLASPTMMHD
6. BjNCED6	282	5.45e-24	LFALSYDVVK	KPYLKYFRFSPEGEKSPDVEI	PLASPTMMHD
3. BjNCED3	334	2.63e-23	LFALSYDVVS	KPYLKYFRFASDGQKSPDVEI	PLDQPTMIHD
4. BjNCED4	310	3.04e-23	LFALSYDVVS	QPFLKYFRFSPDGIKSPDVEI	NLDQPTMMHD
19. BjNCED19	298	5.85e-23	LHALSYDVVK	KPYLKYFRFSPDGVKSPELEI	PLETPTMIHD
16. BjNCED16	298	5.85e-23	LHALSYDVVK	KPYLKYFRFSPDGVKSPELEI	PLETPTMIHD
15. BjNCED15	309	1.84e-20	LFALSYDVVS	KHYLKYFRFTSNGEKSPDVEI	PLDGPTMIHD
24. BjNCED24	304	7.13e-18	ETFAFRYSPV	PPFLTFFRFDSTGKKQRDVPV	YSLTSPSFVH
10. BjNCED10	303	7.13e-18	ETFAFRYGPV	PPFLTFFRFDSTGKKQRDVPV	YSLTSPSFVH
18. BjNCED18	308	1.24e-17	ETFAFRYGPV	PPFLTFFRFDSTGKKQKDVPV	FSMTSPSFLH
21. BjNCED21	307	1.34e-17	LHTLSYNVLK	KPHLKYLKFDTCGKKTRDVDI	TLEQPTMIHD
9. BjNCED9	308	1.70e-17	EMFTFGCSPM	PPYLTYRVISKDGIMHDPVPI	TISKPVMMHD
23. BjNCED23	310	7.77e-17	ETFAFRYGPV	PPFLTFFRFDSTGNKRKDVPV	FSMTSPSFLH
13. BjNCED13	237	8.37e-17	EMFTFGYSQT	PPYLTYRVISEDGFMHDPVPI	TISEPIMMHD
12. BjNCED12	281	8.37e-17	EMFTFGYSHM	PPYLIYRVISKDGIMHDPVPI	TISEPIMMHD
5. BjNCED5	325	9.02e-17	LHTLSYNVLK	KPHLKYLKFDTCGTKTRDLDI	TLEQPTMIHD
20. BjNCED20	565	1.75e-16	EMFTFGYSQT	PPYITYRVISEDGIMHDPVPI	TISEPVMMHD
17. BjNCED17	9	4.03e-10	MASTSSAS	RPHLKSLSFSSSSTLRNKKPT	PCSVSYPVNK
